# Commentary: Arguing for Adaptive Clinical Trials in Sepsis

**DOI:** 10.3389/fimmu.2018.02507

**Published:** 2018-10-30

**Authors:** Miriam Kesselmeier, André Scherag

**Affiliations:** ^1^Research Group Clinical Epidemiology, Center for Sepsis Control and Care, Jena University Hospital, Jena, Germany; ^2^Institute of Medical Statistics, Computer and Data Sciences, Jena University Hospital, Jena, Germany

**Keywords:** sepsis, adaptive clinical trials, Bayesian statistics, platform trials, response adaptive randomization

Adaptive trial designs provide the opportunity to modify design elements during trial conduct. This approach possibly reduces resources, trial duration as well as sample size and may increase the probability of identifying an effective treatment (1). Currently, they are more frequently applied by industrial funders as indicated at clinicaltrials.gov. In a mini-review, Talisa et al. (2) proposed such designs to face the challenges of treatment development in sepsis and underlined their advantages. Finally, they pointed to some challenges in their application. For example, both extensive simulations for the selection of an adequate trial design and statistical models reflecting the trial structure are essential. In general, we completely agree that adaptive designs can be beneficial but they are no magic solutions for all challenges. Moreover, we want to stress that “safety assessments” are not a special topic of adaptive trial designs—data safety monitoring committees will also be in charge here but may require some additional education. With this comment, we would like to initiate a more differentiated view of methodological, statistical and practical challenges related to such designs. We comment on four topics: response adaptive randomization (RAR), adaptive enrichment, seamless, and platform designs.

RAR may be regarded as a kind of futility/inefficiency monitoring because fewer patients are assigned to trial arms in which less efficiency is observed (3). This potentially reduces the total sample size without a considerable loss of precision (1). However, one similarly achieves this aim with adaptive designs without RAR including interim monitoring for futility/inefficiency whose results are easier to comprehend (3). This idea can be extended to flexible multi-arm multi-stage sequential trial designs and, depending on the expected number of effective treatments, applied instead of RAR (4). However, when applying RAR, one must consider further issues. (i) The decision rules for adapting the allocation must be planned upfront of study initiation. Otherwise, introduction of statistical, operational and investigator-driven bias is possible which is difficult to identify and eliminate afterwards (1). (ii) One must pay special attention to the randomization weights update to avoid instable estimates (especially in the trial beginning), extremely unbalanced allocation proportions and erroneous dropping of trial arms (1, 3–6). (iii) Timing plays an important role. First, the outcome might be time-associated, introducing bias if not accounted for (3). Secondly, RAR requires a short-term outcome as otherwise there will be almost no randomization adaptation (1, 7). Short-term outcomes in clinical sepsis research, however, have been questioned (8).

In adaptive enrichment designs, the eligibility criteria are adapted during trial conduct to identify the probably most benefitting patient (sub-) population or the most efficacious dose. This is ideally based on profound understanding of the underlying biology (9). Though, one must decide on the enrichment's primary aim: noise reduction, identification of high-risk patients or of patients most likely showing a positive treatment response (10). Biomarkers are thought to serve as objective indications of biomedical state observed from outside the patient. Roughly one may distinguish prognostic and predictive biomarkers (10, 11). Prognostic enrichment would imply that prognostic biomarker results can be used to increase the selective enrollment of patients having a disease-related endpoint. Predictive enrichment would imply that predictive biomarker results can be used to increase the likelihood of responding to a treatment. Both types of enrichment are of interest for sepsis research but we are facing a “Chicken Egg Problem”: For predictive enrichment there is no biomarker to monitor the response to a sepsis treatment given that we are (still) searching for an effective sepsis treatment.

The idea of seamless designs was introduced in drug development in order to move from phase II to phase III without a recruitment stop while using all the information in a joint final analysis (12). Typically, only a limited number of study arms will enter the phase III stage. The optimal time for the transition from phase II to III or the adaptive changes such as the decision on which arm(s) to drop now become crucial determinants of efficiency (13).

Finally, Talisa et al. (2) talked about platform trials. Generally, the platform idea is basically an IT infrastructure that should lead to a more efficient trial conduct by identifying and selecting patients eligible across many trials in parallel. Incorporation of adaptive design features into the platform design will not only require the introduction of Bayesian statistical models but also a big move in terms of trial logistics. Both topics ask for major changes in the way we teach biostatistics in academic medicine, in the way we recruit patients at our trial centers and on the side of some regulators. We want to make sure that clinicians comprehend the results of Bayesian statistical models, see the use of valid—i.e., cleaned—information for adaptations and finally ensure that robust results get accepted by regulators, e.g., as done with the agreement of the U.S. Food and Drug Administration on an adaptive, seamless phase II/III trial with Bayesian interim analysis (14).

In sum, we agree that changes in clinical sepsis research should also consider changes in the way we run clinical trials. In the short-term, however, we see organizational issues to be addressed first, e.g., the implementation of a central IT platform and a better integration of clinical research into the routine infrastructure [e.g., 15). Regarding the major methodological changes discussed here, costs and benefits need to be carefully checked. Bias is introduced easily and robustness of findings cannot be expected on the base of just a few observations. Frustrating as clinical sepsis trial results were in the past, we nevertheless learned that some of our ideas were likely too simple, including the way that we ran clinical trials.

## Author contributions

Both authors contributed equally to the work, and approved it for publication.

### Conflict of interest statement

The authors declare that the research was conducted in the absence of any commercial or financial relationships that could be construed as a potential conflict of interest.
